# The Strength of Physiological Reports and Peer Review

**DOI:** 10.14814/phy2.13559

**Published:** 2017-12-26

**Authors:** Susan Wray

**Affiliations:** ^1^ University of Liverpool Liverpool UK

To be a founding Editor‐in‐Chief of a journal is always going to be a ride into the unknown, but taking on this role for *Physiological Reports* has been one of the most rewarding activities I have undertaken in my professional life.

When I was appointed, the heavy lifting had already been done by the staff of the two societies that own the journal, The Physiological Society and the American Physiological Society, and our publisher Wiley, leaving me the pleasant task of appointing an editorial team and board, developing and directing editorial policy, and then in April 2013 hoisting a flag up and letting the physiological world know we were open for business.

I am immensely proud of what *Physiological Reports* and the team have achieved in the intervening years. We have published almost 1700 papers so far and attracted authors from 60 countries. We have been joined by the Scandinavian Physiological Society. We do not use perceived impact as a bar to acceptance and so have covered all areas of physiology. Take a look at the papers we have published, and I think like me you will have your belief in the value and the breadth of physiology reinforced. We are seen as the home for those wanting to publish their science in an open access journal, but importantly, a journal that adheres to rigorous ethical and peer view standards. And it is the peer review process that has perhaps surprised me the most during my time with *Physiological Reports*.

As authors we can all point to the deficiencies of the peer review system for our papers; from reviewers who do not recognize our genius and ground breaking work, to those who appear motivated out of less than the goodness of their hearts. As an editorial team we discussed having double blind peer reviewing (i.e., the authors names are hidden from reviewers and vice versa) to having reviewers put their name on reviews, as well as having an open and highly iterative reviewing process. All these and other proposals have been discussed as a mechanism to help fix a broken system, and have vocal supporters.

I have, to my surprise, been reaffirmed in the view that the classical peer review system, with anonymous reviewers, works remarkably well almost all of the time. One quickly learns that one scientist's, “minor revisions” can be another's, “major revisions”; some of us have our glass half full and others half empty. On occasions editors have to decide if a reviewer's recommendation to reject is due to a fatal design flaw, lack of rigor, confidence sapping carelessness and so forth, not seen by the other reviewer, or is because of umbrage taken, hackles raised, and a touch of dyspepsia. The judgments of Solomon have to be made by editors, and it can be a struggle, but unlike the baby in Solomon's choice, not a life or death decision. The overwhelming number of reviews have been constructively critical, fair, consistent, and delivered on time.

I enjoy reading authors' responses to reviewers' comments (sometimes for the wrong reasons!) and seeing how papers are improved via the peer review process. Most authors engage with the comments and appear to be happy with the judgments of their peers and the occasional “good catch,” which will have spared them future blushes. I appreciate that my opinions are on the rosy side, partly because the policy of *Physiological Reports* is to work with authors to get good science published; we are not looking for reasons to reject papers, due to concerns about printing costs, workload, and trendiness of the field or judgments around impact of the paper. Incidentally, very few of our authors take up the option of requesting their work is not sent to certain people. Could this be because we want our rivals to know what we are doing and experience what their thoughts are on it, and have faith in the peer review process?

Getting reviewers for papers, especially those in small fields, can undoubtedly be a challenge, and even an Editor‐in‐Chief only has so many friends and favors she can draw on. Technology however has helped. *Review Locator* is a proprietary platform for text analysis and entity mapping that suggests potential reviewers for each paper. I like this because not only is it helpful, it is also free from biases, as it is gender and geography neutral. We are also using and evaluating Publons, one of several platforms designed to give credits to those who review.

The task of establishing a journal and its processes from the ground upwards was a learning curve for all of us on the team:, Tom Kleyman (Deputy Editor‐in‐Chief), and Associate Editors Julian Davis, Gareth Leng, Meena Rao, Robert Semple, Larissa Shimoda and Morten Thomson, and the 100 strong editorial board, without whose wisdom, hard work, and patience the journal would not have succeeded. From the societies I need to warmly thank all their publications staff, but especially their leaders, Rita Scheman and Simon Rallison. Wiley is fortunate to have excellent staff who have been with us all of the way, Jackie Jones, Jesse Olander, and Fiona Seymour, whose professionalism has been unwavering.

For almost five years I have started most days with a cup of coffee and logged in to *Physiological Reports* – I will have to learn a new routine but am certain the journal will go from strength to strength under its new editorial team. The journal is extremely fortunate to have recruited Tom Kleyman as its new EiC, and I know he will lead *Physiological Reports* to new heights. Submit your paper now!

